# Home-based postnatal midwifery care facilitated a smooth succession into motherhood: A Swedish interview study

**DOI:** 10.18332/ejm/161784

**Published:** 2023-04-24

**Authors:** Margareta Johansson, Li Thies-Lagergren

**Affiliations:** 1 Uppsala University, Department of Women's and Children's Health Akademiska University Hospital, Uppsala, Sweden; 2 Midwifery research - reproductive, perinatal and sexual health, Department of Health Sciences, Faculty of Medicine, Lund University, Sweden

**Keywords:** experiences, home-based care, midwifery, mothers, postnatal care

## Abstract

**INTRODUCTION:**

If a family is discharged from a hospital earlier after birth, close supervision by a skilled midwife is essential. The aim was to describe mothers’ overall experience receiving postnatal care in a Swedish home-based midwifery care model.

**METHODS:**

A descriptive qualitative study was conducted. Mothers meeting the inclusion criteria for a new home-based postnatal care model at a hospital in Stockholm, Sweden, were included. In total, 24 healthy mothers participated in a semi-structured telephone interview, averaging 58 minutes. Data were analyzed using thematic analysis, according to Braun and Clarke.

**RESULTS:**

The main theme explored, ‘The home-based postnatal care model facilitated a smooth succession into motherhood’, is explained by the themes: 1) Mothers felt ‘not left adrift’ when cared for by the home-based postnatal midwives; 2) Professional midwives with authority guided the way into motherhood; and 3) The home, a safe and secure space for new mothers.

**CONCLUSIONS:**

Mothers valued the well-structured home-based postnatal midwifery care. Important for mothers was to receive health checks, adequate information, and that midwives have a kind and individual approach to the families. Midwives play an important role for mothers in the early days after the birth of their baby.

## INTRODUCTION

A shorter hospital requires individualized care, due to stressors and challenges that mothers face during the postpartum period^1^. The reduction of hospital stay should go hand-in-hand with carefully described discharge criteria emphasizing respectful care based on an individual care plan^1,2^. If a family is discharged from maternity care sooner than 48 hours, close supervision by a skilled midwife is vital so that any problems can be prevented or identified promptly, and appropriate intervention or referral can occur^3^. However, many women who give birth are discharged from the hospital within 24 hours after childbirth without information about where they can obtain further care or support^3^, and therefore feel anxious about going home^4^.

Previous research has indicated that mothers who had an early hospital discharge had a positive experience related to the importance of midwives providing sufficient information, support, and guidance to them before the early discharge, to enable them to feel confident to leave the hospital^5^. Mothers in the Swedish context accept well a home-based midwifery care model when discharged early after childbirth^6^. Still, a shared decision on the postnatal care model is a critical but challenging component of maternity care quality. A postnatal care model decision should be influenced by exploring and respecting what matters most for each mother^7^. Important aspects of the mothers’ decision-making process of care model have been noted to be the importance of the time-point for receiving information about the model of postnatal care, to receive sufficient time for consideration about models to choose between as a new mother, to have a rational for choosing a specific care model, and to comprehend the concept of each alternative^8^. However, further knowledge of how mothers experience home-based postnatal care following early hospital discharge is important, as dissatisfaction with postnatal care has been reported^9^. Women and their families are entitled to access models of postnatal care that suit them and ensure adequate support and care. Healthcare providers and health systems must ensure that all mothers receive high-quality, evidence-based, equitable, and respectful postnatal care based on women’s preferences^2,3^. Therefore, the aim was to describe mothers’ overall experience receiving postnatal care in a Swedish home-based midwifery care model.

## METHODS

### Study design and participants

This qualitative study was part of the Postnatal Care Project that aimed to explore mothers, and their partners’ overall experiences of a Swedish home-based postnatal care model^9,10^. Inclusion criteria were: be a Swedish-speaking mother that had participated in the new home-based postnatal care model, had completed a web-based survey about her postnatal experience, and had agreed to participate in an in-depth interview study.

### Study setting

The home-based postnatal midwifery care model was implemented in September 2015 at a hospital in Stockholm, Sweden. A care model was offered to those families meeting the criteria for early discharge (6–24 hours after birth). The home-based postnatal care included daily telephone contact, home visits, and hospital visits, as preferred and needed during the first week after the baby’s birth. The structured follow-up included daily telephone contact by a midwife in the project and an opportunity to call a hospital midwife around the clock if additional support was needed. During the telephone calls, the mothers could book home visits by a midwife as preferred. Previous reports for the project that involved 180 mothers described that they were most likely to have been discharged between 6 and 12 h after childbirth (56%). Most mothers had a positive postnatal care experience from the postnatal model of midwifery care (mean VAS score=8.74, SD=1.438)^6^.

### Data collection

An evaluation of the Postnatal Care Project was initiated 18 months after implementation. In total, 180 mothers completed a questionnaire, and of these, 69 mothers indicated that they could be contacted for a follow-up interview. A total of 35 mothers were contacted via email/short text messages to confirm interest in participation, and 24 mothers accepted to participate in a telephone interview. The qualitative semi-structured audio-recorded telephone interviews were conducted between April 2017 and September 2017 by the first author at a time and day preferred by the participants. The interviews followed an interview guide (Table 1). The participants were encouraged to be themselves and be in a friendly atmosphere. All participants talked freely, and no one withdrew their participation. During the last interviews, the data started to repeat itself, which was understood as a sign of saturation, and the decision was made to terminate the data collection. The length of the interviews varied between 27 and 114 minutes (mean: 58 min). Data collection was carried out between 5 and 18 weeks postpartum (mean: 11 weeks).

**Table 1:** Questions for the interview guide

*No.*	*Questions*
1	How did you experience postnatal care during the first week after the birth of your baby?
2	Describe your sense of participation in the care offered?
3	Please, describe if there are any differences or meanings if a parent receives postnatal care at home or in the hospital after birth of the baby?
4	What does postnatal care imply to you?

Both authors have extensive experience in qualitative methods. The study participants had not met or been cared for by the authors during their postnatal care period. The first author was the project leader who implemented home-based midwifery care.

Background variables were age, civil status, education level, parity, time of hospital discharge after birth (6–12; >12–24; >24 hours) if the discharge was regarded at the right time (Yes; No, I had been able to go home earlier; No, I needed a longer stay), overall experiences of birth and postnatal care satisfaction (measured on a Visual Analogue Scale (VAS), from 0=very negative to 10=very positive), and preferred model of postnatal care for next child (home-based, hotel-based, traditional hospital-based, do not know).

### Data analysis

The data were analyzed using thematic analysis, according to Braun and Clarke^11,12^. The first author familiarized herself with the data, which included transcribing data verbatim, reading and re-reading the data, and noting initial ideas. From the second phase, together with the other author, included generating initial codes with relevant features according to the aim and collating data relevant to each code. Codes were sorted into the nine potential themes:

- Not to be ‘left adrift’
- Return visits for the baby
- Time for talk
- The midwife as an anchor
- Midwifery support for adapting to the parenting role
- Sincere breastfeeding support
- A perfect model when childbirth has gone well
- Home sweet home
- Midwifery care on the family’s terms.

The potential themes were reviewed and collapsed into three new preliminary themes:

- Communication between the family and the postnatal care organization
- Competency and care from the heart
- Importance of being offered alternative care.

The ongoing analysis explored three new themes that were explained by the main theme: ‘The home-based postnatal care model facilitated a smooth succession into motherhood’. The last phase of the data analysis was to include a selection of vivid, compelling quotes in the study’s findings^11^ (Table 2).

**Table 2:** Background characteristics of the study participants (N=24)

*Characteristics*	*n (%)*
**Age** (years), mean (range)	34 (28–41)
**Country of birth**	
Sweden	22 (91.7)
Other	2 (8.3)
**Civil status**	
Married/cohabiting	24 (100)
**Education level**	
High school	3 (12.5)
College/university	21 (87.5)
**Parity**	
Primiparity	1 (4.2)
Multiparity	23 (95.8)
**Time of hospital discharge after birth** (hours)	
6–12	16 (66.7)
>12–24	8 (33.3)
**Experienced discharge at the right time** (as preferred)	
Yes	22 (91.7)
No, I had been able to go home earlier	1 (4.2)
No, I needed a longer stay	1 (4.2)
Overall birth experience*	
10	6 (25.0)
9	7 (29.2)
8	7 (29.2)
7	3 (12.5)
6	1 (4.2)
**Overall postnatal care satisfaction** (VAS)*	
10	8 (33.3)
9	6 (25.0)
8	7 (29.2)
7	3 (12.5)
**Preferred model of postnatal care for next child**	
Home-based care	20 (83.3)
Hotel-based care	2 (8.3)
Traditional hospital-based care	1 (4.2)
Do not know	1 (4.2)

* Score: 0=very negative to 10=very positive. VAS: Visual Analog Scale.

### Ethical considerations

Participation in the study was voluntary, and informed consent was collected from all participating mothers before the interviews started. All participants were informed about confidentiality and that they could withdraw their participation anytime. The study received ethical approval from the ethics committee in Stockholm.

## RESULTS

### Description of the study participants

The mean age of the 24 participants was 34 years. One woman was a first-time mother, and the other mothers had 1–4 children. All mothers lived with their partners and were more likely to have a Swedish origin and higher education. The mothers were discharged between 6 and 24 hours after the birth of their baby, and most of them (91.7%) believed that they had been discharged at an appropriate time. Of the women, 33.3% rated their overall postnatal satisfaction as very positive (VAS score=10). Twenty (83.3%) of the 24 mothers stated that they preferred home-based postnatal care for the next child (Table 1).

### Mothers felt ‘not left adrift’ when cared for by home-based postnatal midwives

A structured follow-up involved a smooth succession into motherhood and included frequent contact with professionals during the first week after the baby’s birth. The follow-up included assessments of the baby’s and the mother’s well-being. The mothers felt taken care of and not ‘left out’. A sense of security was experienced, and they were not ‘left to their own devices’. The mothers described that the home-based postnatal care concept contributed to a good start in motherhood.

To call a midwife directly without going through a switchboard if they had questions was appreciated by the mothers. It was perceived as easy, positive, as extra security, and nice that a midwife was available for them. The mothers missed that the midwives in the project could not take the calls even at night, when instead, an ‘unknown’ midwife at one of the postnatal wards answered who was not fully familiar with the families’ needs and not always documented the telephone call. When the project midwife called the mothers daily during the first week after the baby’s birth to ask for the family’s wellbeing, they experienced the contact as adequate, good, safe, and nice. It was appreciated not to have the responsibility to make the contact themselves. A mother with previous children explained:

*‘I think that is great and a “must”, many mothers will probably not call themselves.’* (Participant 3)

The mothers thought it was easy to ask questions via phone calls. When the midwife made the call, they felt that they did not disturb the midwives as when they had made the contact themselves. One mother was not satisfied with the telephone contact as it was experienced as short and impersonal.

A home visit gave the mothers additional support, a midwifery assessment of the baby’s well-being, and getting help with breastfeeding issues. It was an occasion when the family easily could ask their questions and get help based on their home conditions. The home visits were experienced as nice, natural, pleasant, good, and luxurious, a major bonus that gave them security. Receiving home visits was also appreciated because they did not have to worry about transportation to the hospital for return visits. One mother of three children expressed:

*‘It felt very luxurious to have a person to come home to us, one who could adapt all the information tips and tricks to our home situation.’* (Participant 23)

The mothers appreciated being offered a check-up without having to pre-book an appointment. An assessment that gave the confidence to check if everything was normal. A mother of four children said:

*‘A focus on medical issues is super important, but I think, it’s just as important to focus on mental health aspects and I was asked “Do you feel depressed?”.’* (Participant 3)

The mothers thought that the midwives in the project had an adequate focus on breastfeeding, which was necessary as breastfeeding was described as demanding and fills the hours around the clock, especially during the first week after the baby’s birth. The mothers were usually satisfied with breastfeeding support, which was perceived as valuable. A mother of two children said:

*‘She was just about to leave, so she turned in the door and sat on the edge of the bed to give me tips on lying down during breastfeeding. I thought that it was so nice that she took the time to show. I was very moved.’* (Participant 1)

One first-time mother wanted to receive practical breastfeeding support, but it was not easy to coordinate this during the home visit (Participant 7). Another mother of two children described that the staff during the hospital check-up did not have the time needed for breastfeeding support and that they gave inconsistent advice (Participant 10).

The structured follow-up included a pediatric examination and a hearing screening at the hospital for the baby when needed. The project midwives arranged for the follow-up at the hospital and met the family upon arrival. The return visit to the hospital was perceived by most mothers as good, smooth, flexible, fast, and efficient. The mothers thought this was good and positive; they were satisfied and experienced safety. Not all mothers received information about what the pediatrician assessed. One woman with two children described what information would have meant to her:

*‘You will have a better understanding if you are aware of what they are looking for. If receiving information, you would be better involved, but I still felt that I trusted their skills and I felt safe.’* (Participant 12)

### Professional midwives with authority guided the way into motherhood

Mothers experienced the midwives as being well-read, having a clear plan of action, experienced, skilled, and professional, which were considered positive, good, and safe. Midwives’ health checks and adequate information created safety. The mothers thought of it as good, positive, nice, and safe that the midwife, at an early stage, checked and attended to something that was not right with the baby, the mother, or the breastfeeding. The mothers appreciated that the babies’ weight was examined, that the general condition was assessed, and that newborn jaundice was checked, as well as receiving information about how to nurse the baby. It was important for the mothers to get information about the baby’s health, which led to them being calm if everything was fine.

When the mothers felt listened to, received good, clear, well-adapted information, and received correct and nice treatment, they experienced participation in postnatal care. This approach led to the midwife and the mother meeting on equal terms in the conversations and that the mothers did not feel overlooked or that anyone ‘spoke above their heads’. A second-time mother explained:

*‘They [the midwives] advised me to listen to my instincts. They trusted that I could make my judgment.’* (Participant 1)

Receiving information about the psychological aspects of childbirth was considered important. It was described how some midwives mostly focused on the baby, which was perceived as good, but also that midwives could lack a deeper focus on the mother’s well-being. One mother of four children described the difficulties of explaining oneself as a mother:

*‘It feels like you’re taking up too much space if you talk about yourself. It’s very crucial how you talk about sensitive things with a mother.’* (Participant 3)

The mothers thought it was important, friendly, and safe that the midwife answered their questions. Furthermore, it was appreciated when the midwife asked questions about breastfeeding, how everyone in the family was feeling, and their family’s life situation, to discover if additional support was needed. The mothers appreciated when the midwife offered conversations about their birth experience, listened to the mothers and took them seriously, and when their partner became involved in the conversation.

The midwives asked questions about what the mothers knew, and with this approach, the mothers perceived themselves as participatory, competent, and safe, and became involved in influencing their postnatal care. A mother of three children said:

*‘They didn’t check bits and pieces, never gave lots of opinions, they just asked questions about what I knew. It was a very good approach and made me feel that I was the one who was competent.’* (Participant 21)

The midwives were generally described as not giving their own opinions. But, it was described by one mother of three children how a midwife had expressed her personal opinion about how pacifiers are not needed for a breastfed baby. This was perceived as an unprofessional statement:

*‘She expressed a little personal opinion, and that opinion was not so professionally based, but otherwise, the conversation went well.’* (Participant 16)

Kind and responsive midwives were appreciated, and mothers experienced getting a friendly, pleasant, and nice response from the midwife. The mothers felt that the midwife listened to them and that they cared about the families and wanted the best for them, which created a sense of security in the mothers. Two mothers with previous children described:

*‘Midwives [compared to physicians] have a slightly softer way of seeing everything.’* (Participant 8)

*‘I was treated so well, they were so nice and accommodating. From the beginning, you get a positive experience and become calm.’* (Participant 24).

The midwives were perceived as relaxed and calm, and they worked without stress even though the mothers may have experienced the situation as stressful. The mothers felt that there was enough time for them, even though the midwives most likely very busy. This approach was perceived positively and created a sense of security and a feeling of being strengthened in one’s parental role. A second-time mother described this as:

*‘[The baby] was a little frustrated because he was hungry … [the midwives] were calm in that situation. Had they been stressed by that; we probably would have been even more stressed and wondered if we would have to return to the hospital.’* (Participant 18)

Individual care and support by the same midwife were preferred. The mothers described that they were not checked according to a specific template and were not questioned. They were usually given coherent individualized care and an opportunity to influence their postnatal care. The midwives were described as up-to-date on each family’s situation and needs, which made the mothers feel that they were professional and trusted the mothers’ stories and experiences. The mothers appreciated meeting the same midwife at their contacts with the home-based postnatal care during the first week, which was perceived as very nice and personal. A woman of three children described this as:

*‘I got to see Mary [the midwife] again … it becomes so personal, not like you’re just one in the crowd … and this person knows my story.’* (Participant 21)

Sometimes the mothers met different midwives and could experience it as dreary and impersonal. A mother of three children explained:

*‘It might have been even better [to meet the same midwife] and maybe even more efficient, but I believe the information between these people was so good it didn’t bother me.’* (Participant 23)

### The home is a safe and secure space for new mothers

The mothers experienced feelings such as practical, nice, cozy, fun, and comfortable, the best thing that could happen when receiving postnatal care in their home because they got peace and quiet. They could more easily get into family life, involve any siblings with the newborn baby, and get started with breastfeeding. The home-based postnatal care concept was perceived as practical and flexible for the whole family. One mother with two children said:

*‘This opportunity has been fantastic for us. We were able to return home early and we felt safe with that. Another effect was that we by leaving the hospital we freed up space for others who needed it [better].’* (Participant 2)

Receiving care at home instead of in the hospital was perceived as safe and secure as they could avoid infections such as respiratory syncytial virus both for the newborn baby and for any siblings. Being cared for in a hospital they believed could make them feel sick even though they were healthy.

Being in their home could be perceived by some as not getting adequate rest when they immediately entered their everyday lives. One second-time mother said:

*‘We had a two-year-old at home who also demanded attention and the newborn who wanted my closeness and wanted to breastfeed very often, and I didn’t have time to catch up.’* (Participant 5)

## DISCUSSION

The mothers felt ‘not left adrift’ when cared for by professional midwives with structured postnatal care followup available for them. When mothers know that a midwife is going to visit them at home after an early discharge, their feeling of safety and security may be enhanced^5,6,8^. A professional midwife cares for the childbearing woman and her family, and caring is seen as the core of midwifery for a smooth succession into motherhood^13^. A ‘good’ caring midwife should possess several attributes, such as theoretical knowledge, professional competencies, personal qualities, communication skills, and sound ethical values^14^. Mothers feel safe and supported when the midwives’ approach is balanced when being positive but realistic and not over-idealistic, being encouraging and affirming with an authentic presence^15^.

The mothers did not need to take responsibility to contact the project midwives daily, and they appreciated this procedure. Mothers who are cared for postnatally at home have appreciated that they could call a midwife if there was anything of concern. The midwife’s availability resulted in less stress, and the woman did not need an additional hospital check-up^16^. The mothers described how they missed that the midwives in the project could not take the calls at night, as an unknown midwife who answered the call was not entirely familiar with the family’s needs. Dahlberg et al.^16^ describe that contact with other midwives, other than those who carry out home-based care, often gives the mothers contradictive advice and, therefore, the mothers do not get a response to their worries as needed.

The mothers appreciated meeting the same midwife during the home-based postnatal care. Meeting an unknown midwife during postnatal care has made mothers hesitate to initiate a dialogue, even if the midwife encouraged a dialogue by asking if there was anything of concern^16^. When mothers have a personal relationship with the midwife responsible for the home visit, they are more likely to experience predictability, availability, and confidence in the care given^16,17^.

The home visit was an occasion when the family could more freely ask their questions and get well-adapted practical help. When midwives set aside sufficient time for home visits and take time to answer questions and provide an opportunity for mothers to talk freely about issues of concern to women, they have experienced the professional advice as consistent^5,16^. When mothers receive clear and adequate information, they and their families are satisfied with the postnatal care given^4^.

The mothers described a need to ask for recognition if ‘everything’ was normal, which led to them being calm if everything was ‘fine’. Women often feel vulnerable, emotionally unstable, and insecure during the early days after the birth of their baby because of challenges with their maternal role^6^. During this time-period, mothers have been described as in need of confirmation that everything is normal with themselves and their babies^18^. Being cared for by a kind and professional midwife, provided a positive postnatal care experience for the mothers in our study (Table 3).

**Table 3:** Main theme with themes and sub-themes explored

			
**Main theme**	The home-based postnatal care model facilitated a smooth succession into motherhood		
**Themes**	Mothers felt ‘not left adrift’ when cared for by the home-based postnatal midwives	Professional midwives with authority	The home, a safe and secure space for guided the way into motherhood new mothers
**Sub-themes**		Midwives’ health checks and adequate information created safety	
		Kind and responsive midwives were appreciated	
		Individual care and support by the same midwife were preferred	

To be individually cared for by a professional midwife who trusts and has knowledge about the mother’s circumstances was highlighted as important for postnatal care satisfaction. The International Confederation of Midwives emphasizes the importance of developing a partnership with women that leads to an individual plan for each childbearing woman^19^. The core of healthcare ethics includes the principle of respect for autonomy with the values of individual freedom and choice^20^. Women-centered care is based on recognizing, acknowledging and respecting the childbearing woman with her distinctive needs, ideas, thoughts, emotions, expectations, and wishes about motherhood^21^.

The home-based postnatal care concept was perceived as practical and a flexible solution for the whole family, according to the mothers in our study. Mothers who had previously been given the opportunity to plan their home-based postpartum care with greater flexibility, experienced that the care could be tailored to them and their families by their dynamic physical and emotional needs^22^. Also, the home environment facilitated fathers feeling safe, enhanced their ability to support the woman, and facilitated a fatherinfant bond^10^.

The mothers valued receiving postnatal care in their home environment because they had peace and quiet. Previously, mothers who had early postnatal hospital discharge had a positive experience^5,8^ related to the peaceful and calm home environment^10^. When mothers are discharged early from the hospital, they develop their parental routine more quickly, which increases the sense of control over their day-to-day lives^5^. Mothers wish to return home quickly after the birth of their baby, in order to stay with their family in familiar surroundings^16^. Mothers in Sweden have previously preferred early discharge with home-based midwifery care when having previous experiences of motherhood, mother and baby in good health, and social support available from their family. The home visits were appreciated because the mothers received adjusted breastfeeding support according to their home conditions^10^.

### Strengths and limitations

This study provided in-depth and detailed information^23^ about mothers’ experience of receiving home-based postnatal midwifery care. A strength of this study was the lengthy interviews rich in content. Data were collected by telephone interviews, a method considered convenient as informants could decide for themselves the time of the interview and attend the interview without having to travel^24^. Nevertheless, negative consequences of telephone interviews may be that participants being interviewed through the telephone might be less cooperative than during a face-to-face interview and that body language is missed out^25^. Furthermore, all responders but one had previous experiences with hospital-based postnatal care and were asked to elaborate on the pros and cons of different care models. However, an RCT is preferable when aiming to compare and evaluate different models of postnatal care. Further limitations were that the participants were mainly multiparas, mothers who lived with a partner, had an uncomplicated pregnancy and a vaginal birth and a healthy baby, which should lead to caution of transferring the findings to other groups of new mothers. Beneficial was that most of the mothers had been allowed to choose between either home- or hospital-based postnatal care^6^. Both authors have extensive experience in qualitative methods. The study participants had not met or been cared for by the authors during their postnatal care period. The first author was the project leader who implemented home-based midwifery care. The Standards for Reporting Qualitative Research were used to ensure the transparency of our study^26^.

## CONCLUSIONS

The implemented home-based postnatal midwifery model of care facilitated a smooth succession into motherhood. The mothers felt ‘not left adrift’ when receiving the structured postnatal care follow-up after an uncomplicated childbirth. The model was valued and an acceptable and desired option for mothers. Midwives’ health checks and sharing of adequate information created safety. Important for mothers was to be treated individually by a kind and known midwife that had time for talk and was professional and personal but not private. Mothers regarded their home as a safe and secure space for the whole family. Midwives play an important role for mothers in the early days after the birth of their baby.

## Data Availability

The data supporting this research are available from the authors on reasonable request.
