# Corrigendum: Home-based postnatal midwifery care facilitated a smooth succession into motherhood: A Swedish interview study

**DOI:** 10.18332/ejm/169096

**Published:** 2023-07-24

**Authors:** Margareta Johansson, Li Thies-Lagergren

**Affiliations:** 1 Uppsala University, Department of Women's and Children's Health Akademiska University Hospital, Uppsala, Sweden; 2 Midwifery research - reproductive, perinatal and sexual health, Department of Health Sciences, Faculty of Medicine, Lund University, Sweden

**Keywords:** experiences, home-based care, midwifery, mothers, postnatal care

Corrigendum on:

Home-based postnatal midwifery care facilitated a smooth succession into motherhood: A Swedish interview study

By Li Thies-Lagergren and Margareta Johansson

European Journal of Midwifery, Volume 7, Issue April, Pages 1-8, Publish date: 24 April 2023

DOI: https://doi.org/10.18332/ejm/161784

In the originally published version of the article, the sequence of the authors’ names was incorrect and is now corrected to Margareta Johansson and Li Thies-Lagergren. The order of the affiliations has accordingly changed so that the first affiliation listed is Uppsala University, Department of Women's and Children's Health, Akademiska University Hospital, Uppsala, Sweden, and the second is Midwifery research - reproductive, perinatal and sexual health, Department of Health Sciences, Faculty of Medicine, Lund University, Sweden. The mentioned changes are corrected also online.

